# Applications of Advanced Imaging for Radiotherapy Planning and Response Assessment in the Central Nervous System

**DOI:** 10.3390/tomography11060068

**Published:** 2025-06-12

**Authors:** Liam S. P. Lawrence, Rachel W. Chan, Amit Singnurkar, Jay Detsky, Chris Heyn, Pejman J. Maralani, Hany Soliman, Greg J. Stanisz, Arjun Sahgal, Angus Z. Lau

**Affiliations:** 1 Department of Medical Biophysics, University of Toronto, Toronto, ON M5G 2C4, Canada; liam.lawrence@mail.utoronto.ca (L.S.P.L.); stanisz@sri.utoronto.ca (G.J.S.); 2 Physical Sciences, Sunnybrook Research Institute, Toronto, ON M4N 3M5, Canada; rchan@sri.utoronto.ca; 3 Medical Imaging, Sunnybrook Health Sciences Centre, Toronto, ON M4N 3M5, Canada; amit.singnurkar@sunnybrook.ca (A.S.); chinthaka.heyn@sunnybrook.ca (C.H.); pejman.maralani@sunnybrook.ca (P.J.M.); 4 Department of Radiation Oncology, Sunnybrook Health Sciences Centre, Toronto, ON M4N 3M5, Canada; jay.detsky@sunnybrook.ca (J.D.); hany.soliman@sunnybrook.ca (H.S.); arjun.sahgal@sunnybrook.ca (A.S.); 5 Department of Neurosurgery and Paediatric Neurosurgery, Medical University, 20-059 Lublin, Poland

**Keywords:** magnetic resonance imaging, positron emission tomography, central nervous system, glioma, brain metastases, radiotherapy, MR-Linacs, early response assessment, radiation necrosis

## Abstract

Background/Objectives: Radiotherapy for tumors of the central nervous system (CNS) could be improved by incorporating advanced imaging techniques into treatment planning and response assessment. The objective of this narrative review is to highlight the recent developments in magnetic resonance imaging (MRI) and positron emission tomography (PET) for applications in CNS radiotherapy. Methods: Recent articles were selected for discussion, covering the following topics: advanced imaging on MRI-linear accelerators for early response assessment in glioma; PET for guiding treatment planning and response assessment in glioma; and contrast-enhanced imaging and metabolic imaging for differentiating tumor progression and radiation necrosis for brain metastasis treatment. Where necessary, searches of scholarly databases (e.g., Google Scholar, PubMed) were used to find papers for each topic. The topics were chosen based on the perception of promise in advancing specific applications of CNS radiotherapy and not covered in detail elsewhere. This review is not intended to be comprehensive. Results: Advanced MRI sequences and PET could have a substantial impact on CNS radiotherapy. For gliomas, the tumor response to therapy could be assessed much earlier than using the conventional technique of measuring changes in tumor size. Using advanced imaging on combined imaging/therapy devices like MR-Linacs would enable response monitoring throughout radiotherapy. For brain metastases, radiation necrosis and tumor progression might be reliably differentiated with imaging techniques sensitive to perfusion or metabolism. However, the lack of level 1 evidence supporting specific uses for each imaging technique is an impediment to widespread use. Conclusions: Advanced MRI and PET have great promise to change the standard of care for CNS radiotherapy, but clinical trials validating specific applications are needed.

## 1. Introduction

### 1.1. Overview

Medical imaging technologies have advanced substantially over the past several decades in their ability to measure the quantitative characteristics of normal and pathological tissue in vivo. These advances have implications for the future of the therapeutic management of tumors of the central nervous system (CNS), including gliomas and brain metastases. This narrative review highlights the advanced magnetic resonance imaging (MRI) and positron emission tomography (PET) techniques for radiotherapy (RT) planning and response assessment in the CNS.

### 1.2. Gliomas and Brain Metastases

Gliomas are the most common malignant primary brain tumors in adults [1]. According to the 2021 World Health Organization (WHO) classification of gliomas, a specific diagnosis is reached via histology to evaluate the cell type and anaplasia, as well as testing for the presence or absence of molecular markers [2,3]. Through histopathology, low-grade gliomas show cytologic atypia while high-grade gliomas can additionally show anaplasia, mitotic activity, microvascular proliferation, and necrosis [4]. Molecular features can be determined through sequencing, immunohistochemistry, and DNA methylation profiling. In adults, the three types of diffuse glioma are isocitrate dehydrogenase (IDH)-mutant and 1p/19q-codeleted oligodendroglioma (the best prognosis), IDH-mutant astrocytoma (a middling prognosis), and IDH-wildtype glioblastoma (the worst prognosis). Certain features are needed for diagnosis: for grade 4 glioblastoma, IDH wildtype status and at least one of microvascular proliferation or necrosis or telomerase reverse transcriptase promoter mutation or +7/−10 chromosome copy number changes or epidermal growth factor receptor gene amplification; for grade 2 or 3 oligodendroglioma, IDH mutation and 1p/19q codeletion are both required; and astrocytoma requires IDH mutation with ATRX mutation or loss of expression for grade 2 or 3. The additional presence of cyclin-dependent kinase inhibitor 2A/2B homozygous deletion will push the classification to grade 4 astrocytoma even without microvascular proliferation or necrosis. The O^6^-methylguanine-DNA methyltransferase (MGMT) promoter methylation status is also frequently assessed, since the MGMT status in glioblastoma is both predictive of the response to chemotherapy and an independent prognostic variable [5].

The tumor classification is an important factor in the prognosis and subsequent medical management for the patient. The prognosis for low-grade astrocytoma or oligodendroglioma with extensive resection can be 20 years or more [6]. For IDH-wildtype glioblastoma, even with aggressive treatment, the median survival is only approximately 15 months [7]. The treatment for high-grade glioma comprises maximal surgical resection for debulking and to obtain a tissue sample for diagnosis. Greater resection is associated with better survival rates [8]. Since gliomas are invasive, even in the case of gross total resection, an infiltrating tumor is present surrounding the original tumor site. Hence, resection is followed by radiotherapy and concurrent and adjuvant temozolomide chemotherapy [1,7]. The typical treatment plan is 60 Gy in 30 fractions over 6 weeks for patients less than 70 years old with a good performance status. In the elderly or in frail patients, 40 Gy in 15 fractions over 3 weeks is common [1]. The treatment for low-grade gliomas is not standardized, and can comprise surgery, radiotherapy, chemotherapy, IDH inhibitors, or a combination [9,10,11]. For grade 2 tumors, a “watch-and-wait” strategy without surgical resection can be employed, although evidence suggests worse overall survival relative to surgical resection [12].

Brain metastases are secondary cancers that have seeded in the brain from a primary tumor elsewhere in the body. Brain metastasis has been estimated to occur in 20% of patients with cancer [13,14]. The most common primary sites are the breast, lung, and skin (melanoma) [15]. The histology and molecular features of a brain metastasis depends on the primary tumor, but the genetics of a metastasis can evolve independently from that of the primary cancer [16]. The prognosis depends on the primary site and treatment, but median survival ranges from approximately 5 to 15 months [15]. The treatment options for brain metastases include stereotactic radiosurgery (SRS) for oligometastatic disease (~1–3 lesions), whole brain radiation therapy for many brain metastases, and surgical resection, or a combination [17]. A typical whole-brain radiotherapy fractionation schedule is 30 Gy in 10 fractions [17], while stereotactic radiosurgery will comprise 15–20 Gy in a single fraction or 27–32.5 Gy in 3–5 fractions [18]. Radiosurgery alone is preferred without whole-brain radiotherapy when possible because of the reduced cognitive toxicity and superior local control [19]. Chemotherapy was traditionally not in routine use for brain metastases because of the limited penetration into the brain of many chemotherapeutic agents; however, there are now targeted therapies with central nervous system penetration that are allowing the deferral of radiation in selected circumstances [20].

### 1.3. Neuroimaging and Response Assessment

Magnetic resonance imaging (MRI) is used at every stage of treatment for brain tumors, from diagnosis to treatment planning to response assessment. The routine clinical sequences are T_1_-weighted imaging with gadolinium contrast enhancement and T_2_-weighted fluid attenuated inversion recovery (FLAIR). The T_1_-enhancing lesion is interpreted as the ostensible macroscopic tumor for both gliomas and brain metastases. Gliomas are surrounded by microscopic infiltrating disease and vasogenic edema, visible as hyperintensities via FLAIR imaging [21]. Brain metastases are less frequently infiltrative [22], but are still surrounded by FLAIR hyperintensities due to edema.

Following radiotherapy, the progression or stability of the tumor is determined through the Response Assessment in Neuro-Oncology (RANO) criteria for gliomas [23,24] and the RANO-BM criteria for brain metastases [25]. The response is assessed via changes in the tumor size as evaluated with contrast-enhanced T_1_-weighted imaging. For gliomas, bidimensional measurements of the longest diameters of the enhancing tumor are taken, and the product is calculated as a measure of the tumor size. Measurements are acquired at a reference timepoint (historically post-surgery but more recently recommended to be immediately post-radiotherapy) and at follow-up appointments that occur four weeks after radiotherapy completion and every two to three months thereafter. For brain metastases, unidimensional measurements are used instead. An increase in size (25% for gliomas, 20% for brain metastases) corresponds to a progressive disease while a decrease in size (50% for gliomas, 30% for brain metastases) corresponds to a partial response. If the enhancing lesion completely disappears, then the response is said to be complete. Otherwise, the tumor is classified as stable.

### 1.4. Clinical Challenges

**Figure 1:**
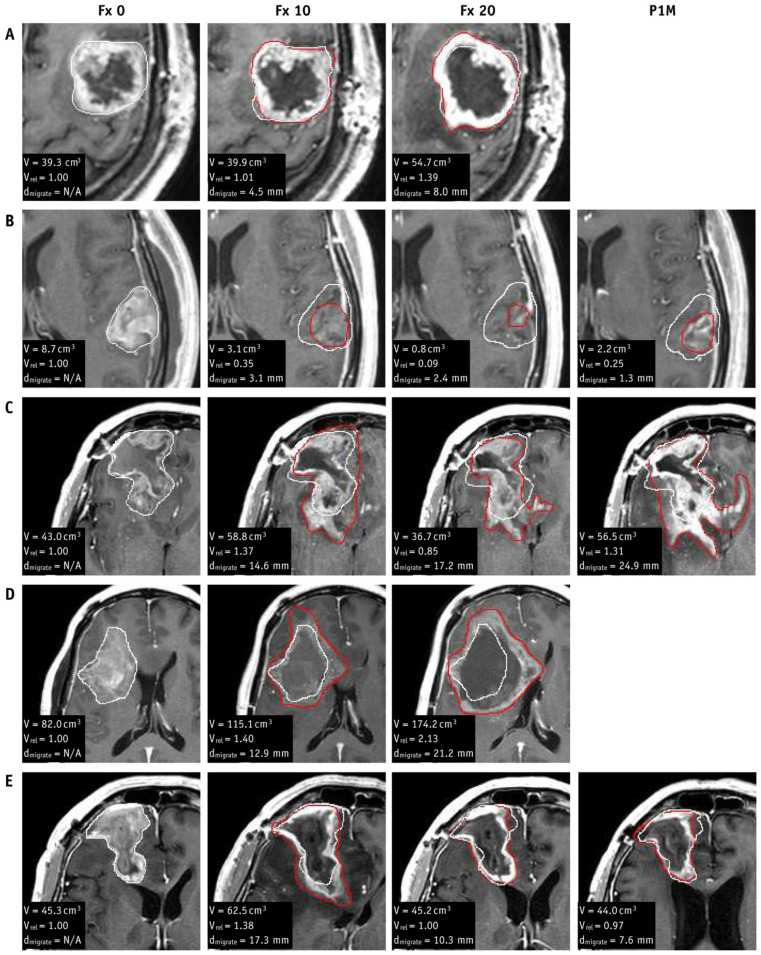
Glioblastomas can change rapidly during treatment. Zoomed images of contrast-enhanced T_1_-weighted MRI for five cases of glioblastomas (**A**–**E**) at treatment planning (Fx 0), fractions 10 and 20 (Fx 10 and Fx 20), and one-month post-radiotherapy (P1M). The white contour shows the gross tumor volume (GTV) at planning while the red contour shows the GTV at the specified timepoint. The absolute volume (V), the volume relative to planning (V_rel_), and the maximum distance of the new GTV from the planning GTV (d_migrate_) are listed in the lower left-hand corner of each image. Figure was reproduced from Stewart et al. with permission from the publisher [26].

There are two limitations of the current radiotherapy strategies for gliomas that will be of relevance later in this review. First, gliomas can change substantially during the three to six weeks of radiation treatment [26,27], but the treatment plan is typically kept unchanged from pre-radiotherapy or is adapted only once. See Figure 1 for examples of changes that can occur in glioblastomas during radiotherapy. Adaptation to ensure tumor coverage may be necessary, especially in the era of small-margin radiotherapy to attempt to spare normal brain toxicity [28,29]. Second, radiotherapy targets are based solely on T_1_- and T_2_-weighted imaging, but enhancing tumor is only reflective of tumor that has disrupted the blood–brain barrier, while T_2_-weighted imaging hyperintensities have a mixture of infiltrating tumor and edema that is difficult to distinguish [21]. Gliomas are spatially heterogeneous in oncogenic signaling, proliferation, and hypoxia [30,31], which renders some regions of the tumor more aggressive or prone to relapse than others. This heterogeneity is not considered for treatment planning.

**Figure 2:**
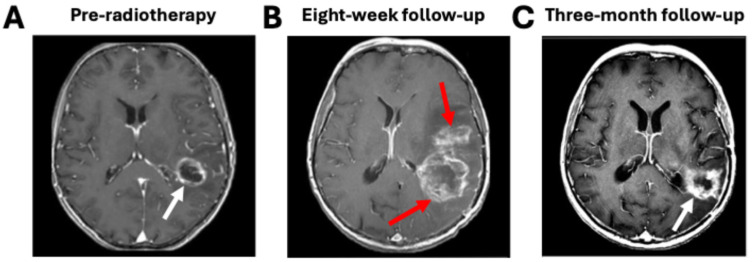
Pseudoprogression mimics true tumor progression. (**A**) A glioblastoma patient immediately pre-radiotherapy shows an enhancing left frontoparietal lesion (white arrow). (**B**) At the eight-week follow-up timepoint, immediately post-radiotherapy, new areas of enhancement are visible (red arrows). (**C**) At the three-month follow-up timepoint, the new regions of enhancement have spontaneously disappeared, and the only lesion visible is at the original tumor site (white arrow). Figure was adapted from Carrete et al. under a Creative Commons License [41].

Response assessment poses another set of challenges common to both glioma and brain metastasis treatment. Although tumor progression is defined as an increase in the size of enhancing lesions, increased enhancement can occur for reasons related to treatment rather than disease. Pseudoprogression is a phenomenon that occurs in approximately 10–30% of patients whereby enhancing lesions develop within the radiation field, sometimes with corresponding clinical symptoms, and then subsequently decrease in size or disappear without intervention (Figure 2) [32]. MGMT promoter methylation predicts a greater chance of pseudoprogression [33]. Pseudoprogression is thought to be related to enhanced vessel permeability resulting from the radiation. Radiation necrosis is another, more severe, treatment effect yielding an increased size of enhancing lesions that involves vascular injury and glial and white matter damage due to the radiation [34]. Radiation necrosis occurs in approximately 5–25% of brain metastasis patients following stereotactic radiosurgery and can be symptomatic and progressive [35]. Because of pseudoprogression, the RANO criteria for gliomas are generally not applied within the first three months after treatment, and the RANO-BM criteria for brain metastases to declare progression can also be delayed [23,25]. Furthermore, changes in macroscopic tumor size are frequently delayed relative to microscopic tumor changes indicative of a response. Preclinical studies have shown that the tumor response to radiation at a cellular level occurs much earlier than changes in tumor size, including changes in metabolism or the onset of apoptosis and necrosis [36]. Clinical studies have demonstrated a correlation between early changes in physiologically sensitive imaging sequences and patient outcomes that are not reflected by macroscopic tumor changes at the same timepoints [37,38,39,40].

### 1.5. Advanced MRI and PET

These clinical challenges could be solved by advanced imaging techniques beyond T_1_- and T_2_-weighted MRI, including “functional” MRI sequences sensitive to tumor physiology and positron emission tomography (PET) with targeted radiotracers. MRI performs imaging by applying radiofrequency (RF) pulses to excite water protons and magnetic field gradients to perform spatial encoding. The RF signal produced by the excited protons is then measured. This process is repeated until sufficient data is acquired to reconstruct an image. Specific combinations of RF pulses and gradients can be used to sensitize the imaging signal to magnetic relaxation times, water diffusion, or other chemical species, all of which can relate to tumor physiology [42]. In PET, a positron-emitting radioisotope is attached to a biologically functional compound which will accumulate wherever that compound is metabolized [43]. The decay of the radioisotope results in the positron annihilating with an electron to produce two photons, which are then captured by a ring of detectors. After enough events are detected, the spatial distribution of the radioisotope can be reconstructed. The abnormal metabolism of tumors can be exploited to design radiotracers that preferentially accumulate in tumor over normal tissue. Various radiotracers exist including glucose analogs for carbohydrate metabolism (18F-FDG), amino acids that are upregulated in membrane transporters of tumor (^11^C-methionine, ^18^F-FET), and nitroimidazole derivatives for detecting hypoxia (^18^F-FMISO) [44].

Advanced MRI and PET imaging methods show the potential to identify more aggressive or metabolically active tumor at the baseline timepoint, or else detect signs of treatment response or resistance early during radiotherapy. These qualities mean that advanced imaging could improve radiotherapy planning or allow early adaptation of the therapeutic strategy. Furthermore, treatment effects such as pseudoprogression or radiation necrosis might be differentiated from true recurrent tumor by measuring the perfusion or metabolism of enhancing lesions.

### 1.6. Structure of This Review

This review aims to summarize the status of specific applications of MRI and PET for radiotherapy planning or response assessment for tumors of the central nervous system. The topics were chosen based on the perception of promise in advancing specific applications of CNS radiotherapy and not covered in detail elsewhere. Where necessary, searches of scholarly databases (e.g., Google Scholar, PubMed) were used to find papers for each topic. The first two sections (Section 2 and Section 3) focus on gliomas, while Section 4 and Section 5 focus on brain metastases. Section 2 reviews advanced imaging on MRI-linear accelerators, which can be used to adapt targets according to the tumor biology. Section 3 reviews the uses of FET-PET for glioma radiotherapy. Section 4 and Section 5 focus on the imaging of radiation necrosis following radiation therapy for brain metastases and differentiation from tumor progression. Perfusion imaging using dynamic susceptibility contrast (DSC) has received much interest for its ability to differentiate between true tumor progression and radiation necrosis [45]. The uses of DSC in neuro-oncology have been reviewed elsewhere [46]; as a result, Section 4 and Section 5 will focus on alternative techniques: contrast-enhanced imaging, the microstructural imaging technique of magnetization transfer (MT), and the metabolic imaging technique of chemical exchange saturation transfer (CEST). See Table 1 for a summary.

The originality of this work lies in the review of multiple imaging contrasts and modalities while focusing on applications specifically within the CNS. We aim to give an accessible overview of the evolving techniques with a focus on imaging methods or applications that may not have been sufficiently reviewed elsewhere. To our knowledge, there is only one other recent review on advanced imaging on MR-Linacs [47], which furthermore was not specifically focused on the CNS. Although systematic reviews and meta-analyses exist for FET-PET in gliomas [48,49], the brief overview provided here is valuable for introducing newcomers to the field. The differentiation between radiation necrosis and tumor progression in brain metastases has been reviewed for contrasts such as perfusion MRI and PET [50,51], but a review of more exploratory yet promising analysis techniques including contrast-enhanced imaging and MT and CEST methods is lacking and is therefore provided here.

## 2. Advanced MRI on MR-Linacs for Adaptive Radiotherapy of Gliomas

### 2.1. MRI-Linear Accelerators

MRI-linear accelerators are devices that combine an MRI scanner with a linear accelerator to allow interfraction and intrafraction adaptation [52]. Since gliomas can change rapidly during treatment [26,27], clinical trials are underway to adapt to tumor shape changes evaluated using T_1_- and T_2_-weighted imaging (NCT04726397, NCT05565521, NCT05720078) [29]. Beyond adapting to structural changes, changes in tumor physiology as measured with advanced MR imaging sequences could be used to assess the response mid-treatment and adapt the dose accordingly [53]. Before using advanced imaging on MR-Linacs in clinical trials to guide the treatment, however, technical and clinical validation must be completed [47,54]. Technical validation includes evaluating the accuracy/bias (difference from reference values) and repeatability (variation between repeated scans) while clinical validation refers to correlating imaging features with tumor biology or patient outcome variables [55].

This section reviews the state of technical and clinical validation for advanced imaging on MR-Linacs which could be applied to guide glioma radiotherapy in the future. Given the focus on the CNS, only studies in phantoms or in the brain will be covered. Brief descriptions of each quantitative technique are given; see the review articles for more details [56,57,58]. There are three commercially available MR-Linacs: the 1.5 Tesla (T) Unity system (Elekta, Stockholm, Sweden), the 0.35 T MRIdian (ViewRay, Oakwood Village, Ohio, USA), and the 0.5 T Aurora (MagnetTx, Edmonton, Canada). The original reports for each MR-Linac are detailed in [59,60,61]. A non-commercial 1.0 T system also exists in Australia [62]. Given that the Aurora system has only been available for a few months as of the time of writing and the Australian system is not commercial, only studies on the Unity and MRIdian systems will be discussed in the coming sections.

### 2.2. Technical Validation: Quantitative Relaxometry

Quantitative relaxometry, or relaxation time mapping, refers to voxel-wise mapping of the magnetic relaxation times including T_1_, T_2_, R_2_ *, and T_1ρ_, which are sensitive to the biochemical environment and structure including myelination, water content, and presence of magnetic ions (e.g., iron) [63]. Kooreman et al. found that in a Eurospin TO5 phantom, T_1_ and T_2_ mapping had fair accuracy, ranging from 2.7 to 14.3% (T_1_) and 10.4 to 14.1% (T_2_) and good long-term repeatability (1.8% for T_1_ and 1.4% for T_2_) [64]. T_1ρ_ sequences have also been demonstrated on the Unity system and show excellent repeatability in agar phantoms (<0.5%) [65]. With a dynamic Look-Locker T_1_ mapping sequence, Tran et al. demonstrated sufficient sensitivity to detect radiation-induced changes in oxygenation in phantoms [66]. Nejad-Devarani et al. showed that the STAGE sequence (STrategically Acquired Gradient Echo, [67,68,69]) could be used to acquire T_1_, R_2_ *, and proton density maps on the MRIdian system [70]. Using an ISMRM/NIST phantom, they showed fair accuracy for T_1_ values (9.5 ± 2.2% bias). Good repeatability was demonstrated for T_1_ and R_2_ * measurements in the phantom (4.7 ± 2.4% and 10.0 ± 9.9%), and in white matter (<1.5%). Park et al. showed that MP2RAGE T_1_ mapping could also be performed on the MRIdian system, showing good accuracy (3.5%) and repeatability (2.2%) in phantoms and excellent repeatability in gray (0.4%) and white matter (0.5%) [71]. Magnetic resonance fingerprinting for the rapid acquisition of relaxation time maps has also been demonstrated on both the Unity and MRIdian and show good accuracy and repeatability in phantoms and feasibility in the brain [72,73].

### 2.3. Technical Validation: Apparent Diffusion Coefficient

The apparent diffusion coefficient (ADC) is a metric derived from diffusion-weighted imaging that quantifies in vivo the diffusivity of water molecules, which is in turn sensitive to the cellular microstructure [74,75,76]. Studies have shown that ADC changes in gliomas correlate with cellularity and outcome in preclinical models and human patients [37,77,78,79]. Kooreman et al. showed that ADC measurements in a NIST phantom have good accuracy (1.9–2.7%) and repeatability (1.7%) [64]. Lawrence et al. showed that ADC values agree between the Unity system and a diagnostic-quality scanner in the brain (white matter bias: −7.3%, gray matter bias: −9.0%) except in cerebrospinal fluid (bias: −20%), possibly because of the lower signal-to-noise ratio on the Unity system [80]. The ADC repeatability in the brain was found to be excellent (white matter, 1.4%; gray matter, 1.8%). Time-dependent diffusion imaging has also recently been explored in phantoms and the brain for improved microstructural characterization [81]. On the MRIdian system, ADC values were found to be accurate (<5% bias) in an ISMRM/NIST phantom by Yang et al. [82]. Given that the typical acquisition of diffusion-weighted imaging uses a single-shot echo-planar imaging (EPI) readout that yields substantial geometric distortion, different sequences have been investigated on both the Unity and MRIdian systems and show that reduced distortion is possible (using turbo spin-echo or multi-shot EPI, for example) without a loss of accuracy and repeatability [83,84].

### 2.4. Technical Validation: Perfusion Imaging

Perfusion imaging refers to sequences that are sensitive to parameters of the cerebral vasculature, changes which have been shown to correlate with patient outcomes [38]. Examples include DSC imaging, dynamic contrast-enhanced (DCE) imaging, arterial spin labeling (ASL), and intravoxel incoherent motion (IVIM) [85,86,87]. Kooreman et al. showed that DCE in phantoms is feasible on the Unity system, albeit with poorer repeatability than other quantitative imaging methods (17.9%) [64]. Intravoxel incoherent motion (IVIM) has been evaluated in the brain on the Unity system, but the repeatability of blood volume fraction measurements (9.0%) was found to be too poor to detect changes in tumors [88]. Arterial spin labeling cerebral blood volume measurements on the Unity system were found to exhibit repeatability similar to that measured in previous studies on conventional MRI (4.4 mL/100 g/min absolute deviation compared to 5.3 mL/100 g/min) [89]. On the MRIdian system, DCE imaging was recently demonstrated by Maziero et al. [90], who showed that a bolus of gadolinium-based contrast agent increased the signal in a flow phantom by 192–252%. Although DSC imaging is the workhorse perfusion imaging technique in neuro-oncology [45], no studies yet exist validating DSC on an MR-Linac, a gap in the literature which should be filled.

### 2.5. Technical Validation: Saturation Transfer

Magnetization transfer imaging methods such as chemical exchange saturation transfer (CEST) and quantitative magnetization transfer (qMT) are sensitive to mobile proteins and peptides and to macromolecules such as myelin, respectively [91,92]. Early signs of a response in tumors may be detectable through changes in CEST signals due to metabolic alterations even before microstructural changes are measurable with ADC [36]. On the Unity system, Chan et al. reported a single-slice CEST sequence and showed an increasing CEST effect for increasing concentrations of ammonium chloride [93]. CEST parameters showed a significant contrast between tumor and contralateral white matter (~10% difference for the magnetization transfer ratio of around ±3.5 ppm). Quantitative magnetization transfer imaging using a 3D balanced steady-state free precession sequence was demonstrated by Tran et al. [94], showing good repeatability and sensitivity to changes in tumors.

### 2.6. Clinical Validation Studies

**Figure 3:**
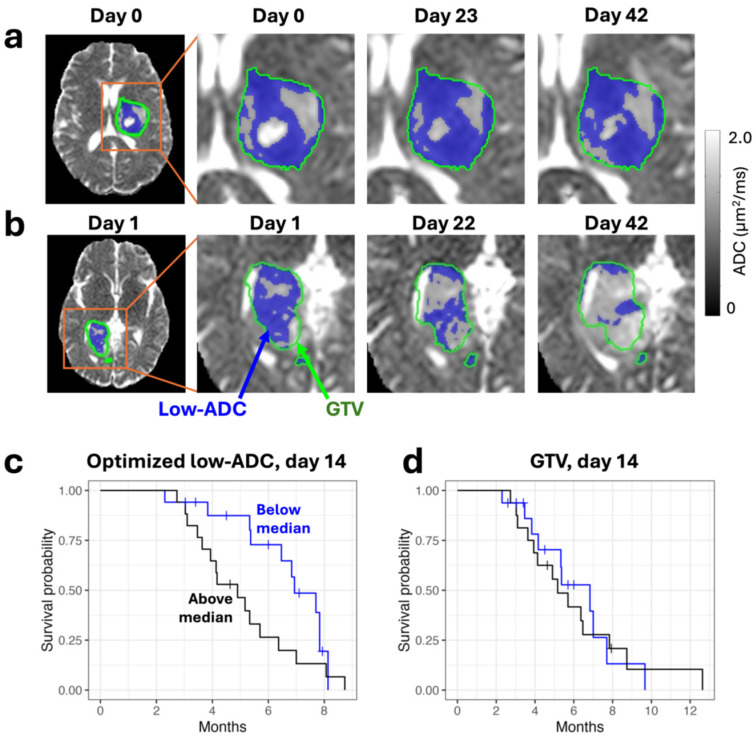
Regions of low-apparent diffusion coefficient (ADC) are correlated with outcome. (**a**,**b**) ADC maps from selected days of treatment on an MR-Linac are shown with overlays of the gross tumor volume (GTV, green contour) and low-ADC regions (blue color-wash). The early progressor (**a**) shows a stable low-ADC region while the late progressor (**b**) shows a shrinking low-ADC region. (**c**,**d**) Progression-free survival with stratification by reference to the change in volume of the low-ADC region and the GTV at day 14 of radiotherapy. The low-ADC region change is correlated while the GTV change is not. Figure was reproduced from Lawrence et al. with permission from the publisher [56].

The number of clinical validation studies is more limited. Lawrence et al. showed that volumetric changes in low-ADC tumor regions (Figure 3), representing presumed dense tumors [95], were found to correlate with progression-free and overall survival from approximately weeks 2–6 of radiotherapy [40,96]. Chan et al. showed differences in CEST metrics between tumor types and grades [93], and also demonstrated that low semi-solid regions of tumors measured with single-slice qMT during radiotherapy precede later tumor progression [97]. Maziero et al. showed on the MRIdian system that the permeability constant (K^trans^) from dynamic contrast enhanced imaging changes in two cases of glioblastoma, and that the changes were consistent with the literature: a 54% decrease was observed in a responder and an 8.6% increase was observed in a non-responder [90].

### 2.7. Summary and Future Directions

The studies just discussed are summarized in Table 2. Many technical validation studies exist; in general, the accuracy and precision of measurements on MR-Linacs are comparable to previous reports from conventional MRI scanners. Further steps for technical development include the greater standardization of imaging sequences, which will enable multicenter studies. Consensus recommendations for ADC acquisitions and processing on the Unity system have been published [98,99], and IVIM acquisition settings also have recommendations [100]. Another direction for technical improvement is creating a dedicated head coil for the Unity system to improve the signal-to-noise ratio, and efforts are underway to create improved coil designs [101]. For other technical developments on the MR-Linac for brain radiotherapy, see also the recent review of Guerini et al. [102].

The next phase of studies should be clinical validation studies relating imaging changes during treatment to clinical outcomes. Importance should be placed on defining volumes from advanced imaging and correlating these volumes with overall survival or with regions of tumor recurrence, so that such volumes can be incorporated into radiotherapy planning and adaptation. Furthermore, proposing adaptive strategies based on advanced imaging and demonstrating their feasibility with respect to dose to the targets and organs at risk should be carried out to advance these techniques to the stage of clinical trials.

## 3. FET-PET/MRI in Gliomas

MRI in combination with PET imaging has been shown to be of importance both in radiation therapy (RT) planning and response assessment/prognostication [104]. One of the first tracers used for the assessment of central nervous system (CNS) malignancies was ^18^F-fluorodeoxyglucose (FDG). FDG is an analog of glucose and is a marker of metabolic activity. While FDG is widely available today, the use of this tracer as a tumor marker is limited due to the brain’s high endogenous glucose utilization due to the high metabolic needs. This limits the utility of FDG as an imaging biomarker for the identification of viable tumors and for the accurate delineation of tumor boundaries.

Amino acid tracers have been shown to be of greater utility than FDG in assessing brain tumors due to their low expression in normal brain tissue and overexpression in both primary and metastatic brain malignancies [104]. Several tracers have been tested in research settings or are available via special access pathways including ^11^C-methionine, ^18^F-fluorodopa, and O-(2-[^18^F]fluoroethyl)-L-tyrosine (FET). Much of the recent literature has aligned around FET due to its favorable physical and imaging characteristics including its physical half-life of 110 min, which allows time for production and transport to imaging centers. Currently, this tracer has received FDA fast-track designation and is at the pre-commercialization stage in the USA; this should lead to more broad availability within the next 3–5 years. Further research and testing are needed with the anticipated incorporation of FET-PET into routine clinical care.

FET-PET as an adjunct to MRI is a compelling value proposition as this provides a comprehensive assessment of both the morphological and functional characteristics of the tumor. For treatment planning, FET-PET has shown that regions of signal abnormality, indicating high amino acid uptake associated with tumor, are often not contiguous with contrast-enhancing tumor on MRI [105,106]. However, the availability of PET/MR scanners are more limited (50 times fewer) than PET/CT [107]. The sparse availability of PET/MRI does not necessarily preclude the use of FET-PET imaging more broadly. PET/CT can still provide value and similar information for brain tumors secondary to advanced co-registration algorithms, which fuse separately acquired PET/CT and MRI and are available from multiple vendors. PET/MR or PET/CT images to aid RT volume delineation have also advanced due to the native ability to create and transfer tumor contour volumes (based on PET information) from a nuclear medicine workstation into radiation treatment planning systems (TPS). This increases the seamlessness and potential routine clinical application of this technology.

The most recent published guidelines from 2021 based on work from the PET/RANO group offer important perspectives on the state of the art and the potential for routine use of PET in radiation oncology [108]. Given the absence of level 1 evidence to support its use, this work acknowledged the requirement of further evidentiary development to introduce this technique into routine clinical practice. In the setting of treatment planning for primary gliomas, the guidelines indicate level 2 evidence for utilization. The use of amino acid PET imaging in the setting of radiotherapy for recurrent tumor is undefined. For the assessment of recurrence, the guidelines reported the availability of level 2 evidence for the aforementioned group of amino acid tracers.

A recent systematic meta-analysis focused specifically on FET was published with findings supportive of its use in both radiation planning and recurrence assessment [48]. Approximately 39% of the patients from three studies on RT planning for gliomas exhibited FET uptake extending beyond a 20 mm margin from gadolinium enhancement, which would inform a material change in RT volumes and is in line with previously published work. For the differentiation of glioma recurrence from treatment-related changes (pseudoprogression and radiation necrosis), the pooled sensitivity and specificity of FET based on the maximum tumor to background ratio was 91% and 84%, respectively. For the differentiation between recurrence versus treatment-related changes in brain metastases, the pooled sensitivity and specificity were 82% and 82%, respectively.

The acceptance of FET as a marker of tumor is based on a foundational neuro-navigated histopathologic sampling study for cerebral gliomas demonstrating a high area-under-the-curve (AUC) for FET-PET/MRI compared to MRI alone (0.98 versus 0.80, respectively) [109]. More recently, in 2023, Harat et al. performed a prospective analysis of over 300 biopsies in 23 patients with adult gliomas wherein biopsies based on FET localization changed the evaluation of the tumor grade in 30% of these patients [110].

Multiple early efforts have been made to close this evidentiary gap, particularly in the setting of target delineation in gliomas based on PET imaging. Despite this early research, amino acid tracers are only available through research trials or special access in a few countries due to the lack of prospective multicenter trials. To address this gap, the Australian-led Trans-Tasman Radiation Oncology Group (TROG) has initiated the FIG trial (ACTRN12619001735145). This multicenter study aims to establish the role of FET-PET in radiotherapy planning and the clinical management of glioblastoma patients [111].

Despite promising evidence, significant barriers remain to the routine clinical adoption of PET imaging in brain tumors. The lack of level 1 evidence is a major hurdle, as current data is largely derived from observational studies and retrospective analyses. Prospective randomized trials are needed to definitively establish the clinical and economic benefits of PET imaging in CNS radiotherapy.

## 4. Contrast-Enhanced Imaging of Radiation Necrosis for Brain Metastases

### 4.1. Conventional MRI

Distinguishing radiation necrosis (RN) from tumor progression (TP) in brain metastases remains a major diagnostic challenge. This difficulty stems from the frequent overlap of RN and tumor tissue on histopathology, which can fluctuate over time and within different areas of the lesion. Consequently, patients often undergo numerous follow-up MRI scans before a definitive diagnosis is reached, and in some cases, a biopsy is required. Improved MRI techniques that can reliably differentiate RN from TP using conventional contrast-enhanced sequences would significantly enhance patient management.

The initial diagnostic strategies for distinguishing RN from TP employed qualitative and semi-quantitative methods, including T_1_-T_2_ mismatch and the lesion quotient (LQ). T_1_-T_2_ mismatch, which refers to the divergence between the enhancing tumor margins on gadolinium-enhanced T_1_-weighted images and the corresponding hypointense tumor margins on T_2_-weighted images, demonstrated a sensitivity of 83.3% and a specificity of 91.1% in a retrospective analysis of 68 patients who underwent stereotactic radiosurgery (SRS) followed by surgical resection [112]. The lesion quotient (LQ), calculated as the ratio of T_2_-hypointense to the contrast-enhancing tumor cross-sectional area, showed promising results in a small retrospective study of 59 patients treated with SRS, where specific LQ ranges were associated with RN and TP [113]. However, subsequent investigations did not reproduce the diagnostic performance of the LQ, resulting in its discontinuation as a reliable method [114].

Contrast clearance analysis (CCA) offers another approach for distinguishing RN from TP [115]. CCA involves generating treatment response assessment maps (TRAMs) by performing image subtraction between T_1_-weighted images acquired at 5 min (early phase) and >60 min (late phase) following gadolinium administration. The resulting TRAMs depict regions of contrast clearance, which are presumed to represent tumor, and regions of contrast accumulation, indicative of RN. In a prospective study involving patients with brain tumors, CCA demonstrated a sensitivity of 93% and a specificity of 78% [116]. Despite its promising diagnostic accuracy, the requirement for prolonged delayed imaging poses a significant practical challenge, potentially limiting its widespread clinical application.

### 4.2. Artificial Intelligence Approaches

Artificial intelligence (AI), particularly machine learning and deep learning (DL), presents a powerful paradigm for extracting subtle, high-dimensional patterns from multiparametric MRI (mpMRI) data. By training algorithms on large, labeled datasets, AI models can learn to recognize the intricate imaging signatures associated with RN and TP, potentially achieving superior diagnostic accuracy and reducing the reliance on subjective human interpretation [117,118]. A recent systematic review and meta-analysis was recently published evaluating the diagnostic performance of machine learning approaches and found a pooled sensitivity and specificity of 77% and 74%, respectively [119]. While these results are promising, they highlight the gap that still exists in the diagnostic performance between machine learning approaches and established methodologies. Currently, few studies have examined the diagnostic performance of DL algorithms and most published results have focused on primary brain tumors rather than metastatic disease.

### 4.3. Contrast-Enhanced T_2_-FLAIR: A New Imaging Biomarker

**Figure 4:**
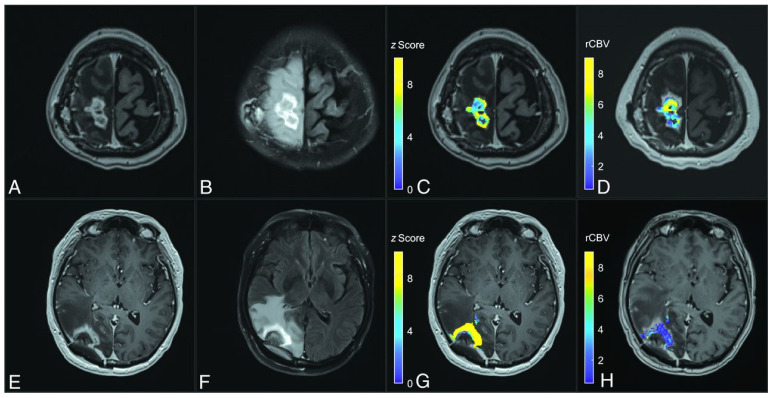
T_2_FLAIR post-contrast (T_2_FLAIRc) z score parameter maps for cases of tumor progression (top row, **A**–**D**) and radiation necrosis (bottom row, E-H). (**A**,**E**) Axial T_1_-weighted MPRAGE post-contrast imaging; (**B**,**F**) T_2_FLAIRc imaging; (**C**,**G**) T_2_FLAIRc z score for enhancing tumor voxels overlaid on T_1_-weighted post-contrast images; (**D**,**H**) the relative cerebral blood volume (rCBV) map from follow-up dynamic susceptibility perfusion MRI. The case of tumor progression shows lower T_2_FLAIRc z values (mean, 5.8 [standard deviation 2.0]) than radiation necrosis (mean, 12.6 [SD 5.7]). The rCBV values are higher in tumor progression (mean, 4.8 [SD 2.3]) compared to radiation necrosis (mean, 1.3 [SD 1.2]). Figure was reproduced from Heyn et al. with permission from the publisher [122].

The existing AI models designed to distinguish RN from TP utilize multiparametric MRI data acquired from standard brain tumor protocols, which commonly encompass diffusion-weighted imaging (DWI), pre-contrast T_1_, T_2_, T_2_-FLAIR, and post-contrast T_1_ [120]. Exploring new imaging sequences and techniques for integration into these protocols represents a promising avenue for improving AI model performance. Contrast-enhanced T_2_-FLAIR (T_2_FLAIRc) has shown the potential to provide additional diagnostic information. At our institution, T_2_FLAIRc is routinely acquired alongside pre-contrast T_2_-FLAIR, primarily to enhance the detection of leptomeningeal disease [121]. Recent investigations have demonstrated that the standardized T_2_FLAIRc signal intensity is significantly elevated in RN compared to TP, suggesting its utility as a novel imaging biomarker (Figure 4) [122]. Furthermore, a univariable analysis using standardized T_2_FLAIRc achieved a sensitivity of 75% and a specificity of 86% in differentiating RN from TP. Based on these promising results, we hypothesize that AI models incorporating T_2_FLAIRc within mpMRI protocols will demonstrate superior performance compared to univariable models. Furthermore, T_2_FLAIRc should serve as a valuable parameter for augmenting the capabilities of deep learning (DL) models.

## 5. Predicting Radiation Necrosis with Metabolic Imaging

### 5.1. MRS, Perfusion, and Non-MRI Techniques

Magnetic Resonance Spectroscopy (MRS) is a key metabolic imaging sequence for brain imaging and has been investigated in brain metastases (BMs) for distinguishing RN from TP [123,124]. Differences in metabolic ratios between RN and TP have been found including those of lactate, creatine, phosphocreatine, and choline-containing compounds [125]. In nine patients with BM treated via Gamma Knife radiosurgery, higher diagnostic accuracy was demonstrated using multi-voxel proton MRS compared to single-voxel proton MRS for differentiation [126]. Intravoxel incoherent motion (IVIM) perfusion has also been shown to differentiate tumor recurrence from RN, and performs better than the apparent diffusion coefficient (ADC) obtained from diffusion-weighted imaging (DWI) [127]. DCE-MRI with quantitative MRI modeling has also been explored for this application in a pilot study [128] which found that changes in model parameters between the pre- and post-treatments scans were correlated to tumor volume changes. Non-MRI techniques such as positron emission tomography [129] and single photon emission spectroscopy (SPECT) [130] have also been investigated and are sensitive techniques for distinguishing TP from RN, but have lower spatial resolution compared to MRI. A retrospective study [131] with 25 patients demonstrated a high negative predictive value of MRS for differentiating recurrent tumor from RN, and showed that MRS has better accuracy than PET/CT. An extensive review of imaging techniques including perfusion MRI, MRS, PET, and SPECT for distinguishing RN from TP can be found in Mayo et al. [50].

### 5.2. Pilot MT/CEST MRI Studies for Predicting RN vs. TP

More recently, chemical exchange saturation transfer and magnetization transfer [132,133,134], which offer higher spatial resolution compared to previous techniques, have been applied in neurooncological applications [135]. CEST and MT show promising results in numerous applications for the treatment of central nervous system tumors [91,136,137,138]. CEST MRI [91,139] can indirectly detect, in low concentrations, exchangeable protons including those of amide groups that exist in endogenous proteins and peptides. CEST is based on protons within metabolites physically exchanging places with other nearby protons, for example, those of water molecules in the so-called “free water pool”.

While the biological origins of the amide CEST signal are not particularly specific (in that it arises, for example, from anywhere in the brain consisting of amide groups in close proximity to water molecules), the CEST signal is sensitive to processes of molecular exchange occurring at the cellular level. The MT signal is more well-defined and reflects brain areas where there are macromolecules—in myelin, for example. Thus, the degradation of white matter due to tumor or necrosis typically manifests as a low quantitative MT fraction compared to healthy white matter. Both MT and CEST are usually run with high and low powers. Using multiple power levels allows for improved parameter estimates. A higher power level provides an image contrast that is more heavily MT- or CEST-weighted.

The application of CEST for differentiating RN from TP in BM patients has been limited to date. The initial pilot studies include Desmond et al., 2017, where quantitative CEST parameters in 25 patients were shown to predict tumor volume changes at one-month post-SRS treatment [140]. Mehrabian et al. [141] showed in 16 BM patients that MT and CEST are capable of differentiating RN and TP, with the best separation achieved by using the magnetization transfer ratio (MTR) metrics at 3.5 and −3.5 ppm.

### 5.3. Differentiation of RN and TP in a Clinical Setting

**Figure 5:**
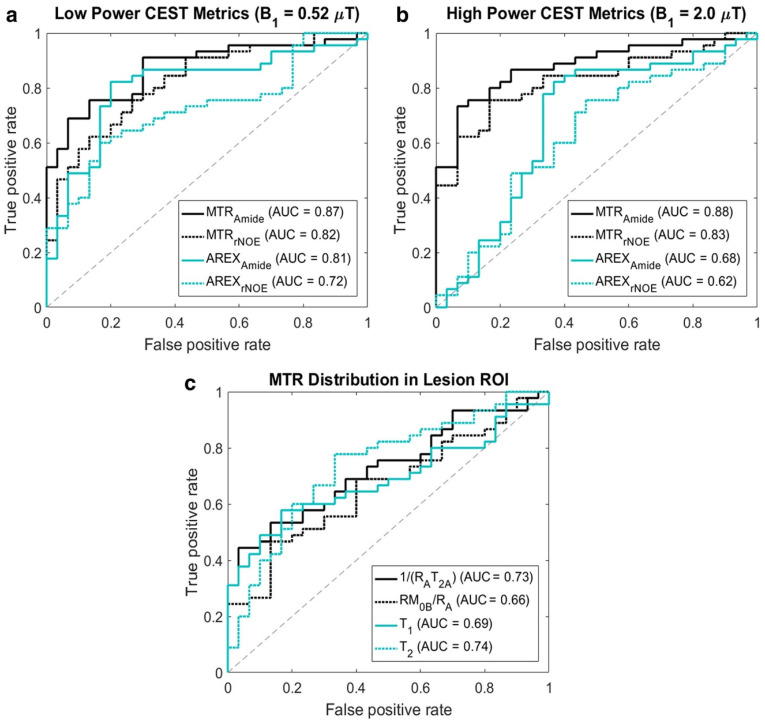
Receiver operating characteristic curves demonstrating the performance of each metric for the differentiation of radiation necrosis from tumor progression for (**a**) low-power chemical exchange saturation transfer (CEST) metrics, (**b**) high-power CEST metrics, and (**c**) magnetization transfer and direct effect metrics. CEST is run at low and high powers for improved parameter fitting, with the higher power corresponding to greater CEST weighting in the image. The CEST metrics (**a**,**b**) are thought to be related to concentrations of proteins and peptides as well as changes due to necrosis, which may explain their ability to differentiate TP and RN. This figure is reproduced from Mehrabian et al. under a Creative Commons License [142].

Later studies of MT and CEST in BM expanded to larger cohorts [142,143], and the methods were tested in a clinical setting. Mehrabian et al. [142] achieved high accuracy for differentiating TP and RN in 70 patients with BM after treatment with stereotactic radiosurgery. This study was conducted using a 2D continuous-wave saturation transfer MRI sequence (i.e., using a long-duration block-shaped RF pulse). The greatest image contrast between RN and TP lesions was found in the MTR parameter at a frequency offset of 3.5 ppm (corresponding to the amide chemical group) using a saturation B_1_ of 2 μT. The AUC of this parameter as a predictor of TP versus RN was 0.88. The receiver operating characteristic (ROC) curves are shown in Figure 5 for the entire set of parameters. Smaller differences were also found in the AREX metric (a parameter used to remove T_1_ and semisolid MT contrast from MTR, to isolate the CEST effect) and several quantitative semisolid MT model parameters. MTR was best for differentiating RN from TP even though it combines multiple effects including the CEST, MT, and direct effects. It was found that MTR asymmetry, although still significantly different between RN and TP, did not perform as well at high saturation powers, which is likely due to it being a noisier parameter compared to MTR.

### 5.4. Pulsed Saturation

In certain MR scanners, a long continuous RF pulse cannot always be implemented due to hardware limitations. In such cases, a “pulsed” (i.e., on and off) approach can be used instead [93,144]. A pulsed saturation technique was explored in a study of 73 BM patients [143] using a train of Gaussian pulses in a 3D MT/CEST sequence for the purpose of differentiating RN from TP. In this study, dynamic susceptibility contrast perfusion MRI was included in a subset of 49 patients. The results showed that the quantitative MT parameter 1/(R_A_·T_2A_), where R_A_ is the 1/T_1_ of the free water pool, and where T_2A_ is the T_2_ of the free water pool, was significantly different in RN vs. TP (with 5.9 ± 2.7 for RN and 6.5 ± 2.9 for TP) using univariable logistic regression. Multivariable analyses showed that using a pulsed technique can achieve an AUC of 75% for differentiating between RN and TP. This study concluded that a continuous-wave, block approach is likely required for higher saturation for improved MT/CEST image contrast. The results also showed that MT/CEST parameters had greater efficacy than relative cerebral blood volume (rCBV) from DSC for differentiating RN from TP.

### 5.5. MT/CEST Maps in a Specific Case of Radiation Necrosis Versus Tumor Progression

**Figure 6:**
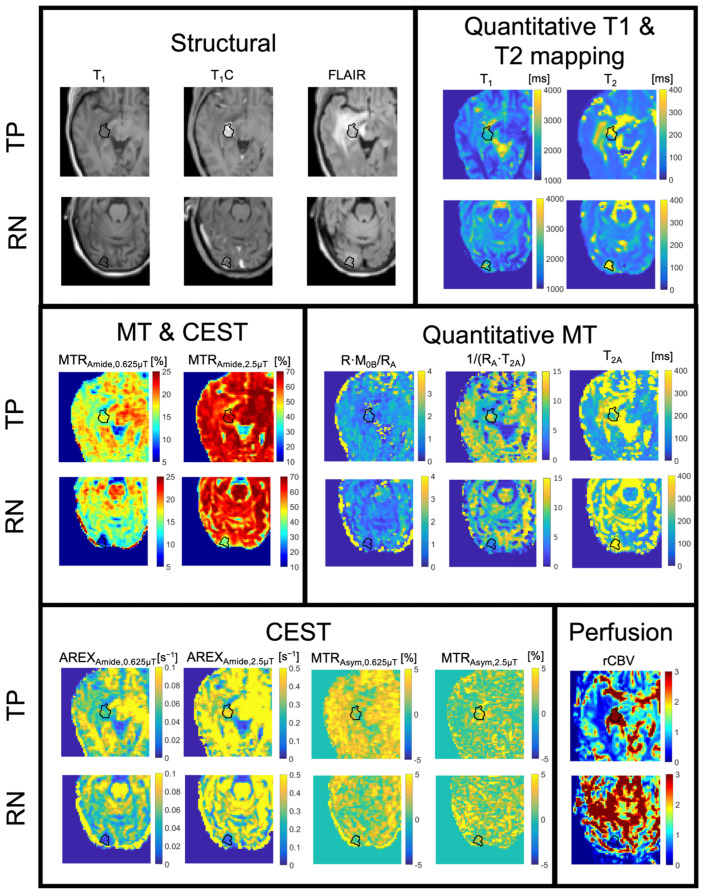
Parameter maps for one example of tumor progression (TP) compared to one example of radiation necrosis (RN). The panels divide images by contrast: structural images (pre- and post-contrast T_1_-weighted and FLAIR), quantitative T_1_ and T_2_ mapping, magnetization transfer and chemical exchange saturation transfer ratios (MT & CEST), and quantitative MT, CEST, and DSC perfusion. Within each panel, the top row shows the case of tumor progression and the bottom row the case of radiation necrosis. The black region-of-interest overlays show the whole-tumor contours for each patient. Patients had prior SRS with radiation doses of 16 Gy in one fraction (TP case) and 25 Gy in five fractions (RN case). This figure is adapted from Chan et al. under a Creative Commons License [143].

Example MT/CEST parameter maps are shown in Figure 6 below, along with the relative cerebral blood volume from DSC, for examples of RN and TP. The set of maps includes structural images (i.e., pre- and post-contrast T_1_-weighted and T_2_-weighted FLAIR images), the observed T_1_ and T_2_ from quantitative mapping, MTR maps consisting of a mixture of MT/CEST/T_1_/T_2_-weighting, MTR asymmetry or “APT” maps (which are derived from the voxelwise subtraction of MTR_Amide_ from MTR_NOE_ maps most commonly used in CEST literature), qMT-fitted maps including R·M_0B_/R_A_ (proportional to the semisolid fraction M_0B_), 1/(R_A_·T_2A_), and T_2A_ (related to the direct effect), and AREX maps that aim to isolate the CEST effect from MT and direct effects [145]. This example of TP showed a hyperintense MTR signal and hyperintense AREX signal compared to normal brain tissue, whereas RN showed hypointense signals. Additionally, the rCBV maps from DSC perfusion showed an elevated signal for TP and hypointense signal for RN.

### 5.6. Origins of the MT/CEST Signal

The precise origins of the MT and CEST signal in RN and TP remain to be determined. However, studies have shown that the CEST signal from necrotic cores is linked to cellular changes in cellular necroses, damaged vessels, and the loss of normal brain tissue [146]. An increased CEST effect has been attributed mainly to higher cytosolic content of proteins and peptides in tumors [146,147] and increased protein expression levels [148], as well as slightly increased amide proton exchange rates [147]. Changes in the MT signal in RN could be related to decreased semi-solid content in necrotic regions, decreased cellularity, or cell membranes. The isolation of the CEST effect from changes in other MRI parameters, such as T_1_ or T_2_, is challenging and is an active area of research. Quantitative CEST may help to separate out the different contributing factors to the signal.

## 6. Conclusions and Future Directions

Advanced imaging techniques have the potential for application in improved radiotherapy targeting, treatment guidance during radiotherapy, and response assessment after radiotherapy completion. MRI and PET can be made sensitive to a variety of physiological processes that can help determine tumor aggressiveness or distinguish cancerous tissue from treatment effects. However, much of the existing research comprises retrospective, single-center studies. This research should be leveraged to design methodologies for prospective, multicenter studies to clinically validate advanced imaging techniques for early response assessment or differentiation of treatment effects and tumor progression.

The imaging techniques that have already been extensively studied should be clinically validated first, including diffusion MRI, perfusion MRI, and FET-PET. To give a concrete example, diffusion MRI could be validated as a technique for early response assessment by selecting a specific timepoint (e.g., 3 weeks) and a specific method of acquisition and analysis (e.g., changes in low-ADC tumor with a threshold of 1.25 μm^2^/ms) that have been established as yielding a strong prognostic variable in an existing study with a large cohort (e.g., [40,95]). This strategy would allow statistical power analysis for the estimation of the required sample size. Prospectively acquired data from multiple centers could then be used to test the correlation with overall survival. Imaging techniques which are earlier in technical development, like CEST and quantitative MT, show great promise in applications for the treatment of CNS tumors, but require further technical studies to understand the biophysical effects that contribute to the signal. More details on the technical and clinical development of imaging biomarkers in oncology, including the requirements for multicenter trials, can be found in the consensus paper by O’Connor et al. [54]. Ultimately, it is hoped that improving the ability to measure aspects of tumor physiology at each stage of treatment using advanced imaging will translate to better management of patients with brain tumors.

## Figures and Tables

**Table 1:** Overview of imaging methods covered in this review article. The “timing” column indicates the timing with respect to the course of radiotherapy for which the application is relevant.

Section	Method	Disease	Application	Timing
2	Advanced imaging on MR-Linacs	Gliomas	Adaptive radiotherapy	During
3	FET-PET	Gliomas	Planning and response assessment	Before and after
4	Contrast-enhanced imaging	Brain metastases	Tumor progression vs. radiation necrosis	After
5	MT and CEST	Brain metastases	Tumor progression vs. radiation necrosis	After

**Table 2:** Studies of technical and clinical validation for advanced MRI sequences with potential use in glioma radiotherapy on commercially available MR-Linacs. Technical validation studies include those evaluating advanced imaging in phantoms; clinical validation studies are restricted to those in glioma patients.

Study	System	Sequence	Level of Validation
Kooreman et al., 2019 [64]	Unity	Relaxation mapping	Technical
Bruijnen et al., 2020 [72]	Unity	Relaxation mapping	Technical
Kooreman et al., 2022 [65]	Unity	Relaxation mapping	Technical
Tran et al., 2024 [66]	Unity	Relaxation mapping	Technical
Park et al., 2024 [71]	Unity	Relaxation mapping	Technical
Kooreman et al., 2019 [64]	Unity	ADC	Technical
Lawrence et al., 2021 [80]	Unity	ADC	Technical
McDonald et al., 2023 [84]	Unity	ADC	Technical
Jokivuolle et al., 2025 [81]	Unity	ADC	Technical
Lawrence et al., 2023 [40]	Unity	ADC	Clinical
Lawrence et al., 2024 [96]	Unity	ADC	Clinical
Kooreman et al., 2019 [64]	Unity	DCE	Technical
Straza et al., 2020 [103]	Unity	IVIM	Prelim. Technical
Lawrence et al., 2021 [88]	Unity	IVIM	Prelim. Technical
Chan et al., 2021 [93]	Unity	CEST	Technical & Clinical
Tran et al., 2023 [94]	Unity	qMT	Technical
Chan et al., 2023 [97]	Unity	qMT	Prelim. Clinical
Lawrence et al., 2024 [89]	Unity	ASL	Prelim. Technical
Nejad-Devarani, 2020 [70]	MRIdian	Relaxation mapping	Technical
Mickevicius et al., 2021 [73]	MRIdian	Relaxation mapping	Technical
Yang et al., 2016 [82]	MRIdian	ADC	Technical
Gao et al., 2017 [83]	MRIdian	ADC	Technical
Maziero et al., 2024 [90]	MRIdian	DCE	Technical & Prelim. Clinical

Levels of validation: Technical, a published journal paper characterizing accuracy and/or precision in vivo; Clinical, a published journal paper correlating imaging with diagnosis or outcome in more than 10 subjects; Prelim. Technical, a conference abstract characterizing accuracy and/or precision in vitro or in vivo; Prelim. Clinical, a conference abstract or journal paper correlating imaging with diagnosis or outcome in 10 subjects or fewer.

## Data Availability

Data sharing is not applicable.
